# “No One Told You Life Was Gonna Be This Way”: A Qualitative Exploration of Friendship Expectations and Reality in University Life

**DOI:** 10.1002/jad.12489

**Published:** 2025-04-04

**Authors:** Michael Priestley, Hannah Rachael Slack, Miss Madiha Islam, Delia Fuhrmann, Emily Long, Sarah Crook, Juliet Foster, Sophie Homer, Nicola Byrom

**Affiliations:** ^1^ School of Education Durham University Durham UK; ^2^ Department of Psychology Institute of Psychiatry, Psychology and Neurosciences, King's College London London UK; ^3^ MRC/CSO Social and Public Health Sciences Unit University of Glasgow Glasgow Scotland; ^4^ Department of History, Heritage and Classics Swansea University Swansea UK; ^5^ School of Psychology, Drake Circus, Plymouth University of Plymouth Plymouth UK

**Keywords:** ADOLESCENT DEVELOPMENT: loneliness, ADOLESCENT DEVELOPMENT: well‐being, ADOLESCENT RELATIONSHIPS: connectedness, ADOLESCENT RELATIONSHIPS: friendship and intimacy, ADOLESCENT RELATIONSHIPS: peer relationships, ADOLESCENT RELATIONSHIPS: socialization and social development, ADOLESCENT RELATIONSHIPS: transitions, GROUP OR ENVIRONMENT: college students, METHODOLOGY: qualitative

## Abstract

**Introduction:**

Young adulthood (ages 18–25) is a high‐risk period for loneliness, particularly during educational transitions. Loneliness has negative consequences for mental health, physical health, and educational achievement. Psychologists conceptualize loneliness as emerging from a discrepancy between expected and experienced social connection, but this has been under‐explored during young adulthood.

**Method:**

Drawing on thematic analysis of eight focus groups with 21 young adults in the UK, this paper explores the differences between retrospective expectations and experience of social connection during the transition into university and the implications for loneliness.

**Results:**

Whilst social expectations, experiences, and preferences vary considerably, young adults' perception of whether expectations are met is ostensibly more consequential for understanding social (dis)satisfaction than objective indicators of the social experience, such as number or quality of friendships. Moreover, discrepancies between social expectations and experience are intensified by a widespread presumption that social relationships in adulthood will form and function as they did at school, resulting in unexpected barriers, challenges, and effort involved in friendship formation.

**Conclusions:**

The findings affirm the importance of addressing loneliness holistically during points of transition and creating socially supportive communities for young adults, particularly at university.

## 1 Introduction

Imagine a university campus, and you might picture a bustling hive of academic and social activity, where, for students, the pull towards societies and social events is just as strong as that towards lectures and coursework. This is the picture that university marketing portrays, a balanced experience with ample opportunities for learning and socializing (Mogaji and Yoon 2019). Yet, up to half of students report experiencing loneliness (Diehl et al. 2018; GOV.UK 2023; Neves and Stephenson 2023).

Loneliness, peaking between ages 18 and 29 (Barreto et al. 2021; Hawkley et al. 2022), is increasingly prevalent among young adults internationally (Buecker et al. 2021; Twenge et al. 2019). For young adults going to university, the peak in loneliness may be exacerbated because they hold high expectations for sociality, that is the degree to which individuals associate in social groups or form societies, at university (Balloo 2018; Hughes and Smail 2015) which often go unmet (Worsley et al. 2021). Few contemporary studies have explored in‐depth the nature of students' expectations for university life, the extent to which they are met, and the consequences if they are not. We seek to elaborate understanding of social experience at a time of educational transition in three ways. First, we place the individual student within their wider social context, considering the social environment that shapes expectations and experience. Second, we consider the developmental factors impacting expectations and experience. Third, we explore the heterogenous dimensions of social expectations.

Around a quarter of undergraduate students report feeling lonely most or all of the time (Buecker et al. 2021; Neves and Stephenson 2023). Whilst transient episodes of loneliness are common during educational transitions (Bosma et al. 2015) these can cause or consolidate chronic loneliness if unaddressed (Wolska and Creaven 2023). Loneliness predicts decreased academic performance, retention, and employability (Bryan et al. 2024), and negative physical (Klaiber et al. 2018) and mental (McIntyre et al. 2018; Richardson et al. 2017; Vasileiou et al. 2019) health outcomes. Loneliness is experienced unequally, with higher levels among minoritised students, potentially driving health inequalities (Frampton et al. 2022; Frampton and Thompson 2023; Living Black at University 2022; Smithies and Byrom 2018; Solís García et al. 2024).

Loneliness remains a complex and contested concept (Stein and Tuval‐Mashiach 2015). There is consensus that humans possess an evolutionary, universal, and “pervasive desire to form and maintain at least a minimum quantity of lasting, positive, and significant interpersonal relationships” (Baumeister and Leary 1995, p. 497). Social relationships can provide guidance, attachment, security, companionship, emotional nurturance and reassurance of worth (Weiss 1984, 1974). The social needs perspective of loneliness (e.g., Diehl et al. 2018) and the developmental model of loneliness (Parkhurst and Hopmeyer 1999; Qualter et al. 2015) distinguish between social and emotional loneliness as a self‐perceived “lack of social relationships” and a “lack of a close, intimate attachment to another person” respectively (Weiss 1974, p.17).

Conceptually, loneliness is distinguished from the objective absence of social relationships, through a cognitive discrepancy approach (Peplau 1982; Verity et al. 2021). Loneliness is understood as the perceived discrepancy between an individual's desired or expected form of social relationships and the individual's actual social relationships (Perlman and Peplau 1981), in terms of relational quantity and/or quality (Qualter et al. 2015). Once placed in these terms, loneliness can be the consequence of a suboptimal quantity or quality of relationships, or higher relational expectations, or both (Marangoni and Ickes 1989).

In the context of rising prevalence of loneliness, a socio‐cultural perspective may provide explanatory value. The discrepancy model of loneliness focuses on the individual, with cognitive theories considering interpersonal expectations (Jones 1982), problem solving (Horowitz et al. 1982), and attributional styles (Anderson et al. 1983). Correspondingly, interventions tend to focus on the individual (Eccles and Qualter 2021; Hickin et al. 2021). However, while it may be an individual who perceives a discrepancy between expected and experienced social connection, a socio‐cultural perspective asks how these expectations are formed. Students' expectations of university life arise from networks of socially shared understandings, not only about university but also about relationships, friendship, and young adulthood (Duveen 2000). These ideas develop, circulate and are maintained within families and friendship groups (microsystem), communities, including university programmes of study and societies (exosystem), and the wider societies (macrosystem) of which students are a part (Bronfenbrenner 1979), and are not only enacted via direct communication and conversation, but also via different forms of media, rituals and practices, policy, and even the very ways in which we organize our societal structures (Flick et al. 2015). The expectation that university will be a social experience is well‐established: students have long seen the opportunity to meet a variety of people as a primary benefit of going to university (Marris 2018). Students draw on these forms of understanding to make sense of their own and others' experience.

The elements of socially constructed expectations that take prominence for students may themselves be influenced by developmental factors. During adolescence and early adulthood, young people spend less time with family and caregivers and more time with friends or alone (Larson and Richards 1991; Larson 1990). The importance of romantic relationships increases too (Collins 2003; Pollmann et al. 2023). Adolescence and early adulthood are characterized by protracted changes in social cognition, including ongoing development in theory of mind, heightened peer influence, and susceptibility to social rejection and acceptance (Albert and Steinberg 2011; Dumontheil et al. 2010; Foulkes and Blakemore 2016; Somerville 2013). Friendships during this time support key developmental tasks such as identity formation and shape well‐being and mental health later in life (Güroğlu 2021).

Elaborating the discrepancy model of loneliness, we seek to explore the heterogenous dimensions of expectations for sociality. Young adults have been found to hold particularly high expectations for frequency of social contact, companionship, and intimacy from friends (Nicolaisen and Thorsen 2017) and expect university to be an exciting new social opportunity (Balloo 2018; Balloo et al. 2017), characterized by strong social connections (Hughes and Smail 2015). There has long been concern about the incompatibility of expectations and experiences of university (Baker et al. 1985; Buckley 1971; Herr 1971; Jackson et al. 2000; Pate 1970; Stern 1966).

Previous research has found that experiencing a mismatch between expected and experienced quantity and quality of relationships enhances feelings of loneliness (Worsley et al. 2021, p.579). However, our friendship networks can vary in stability, size, homophily (tendency to be friends with similar others) and transitivity (friendship via a common other, i.e., friends of friends become friends) (McPherson et al. 2001). We thus seek to understand the dimensions of young adults' expectations for social connection, to better understand how, when, and why mismatches with experiences arise.

## 2 Methods

### 2.1 Participants

Participants were recruited during February 2023 from one large research‐intensive university (King's College London, UK) using email circulars inviting students to share their expectations of university social life as part of a self‐selective convenience sampling strategy. All participants had to be current first year undergraduate students at King's College London. There were no exclusion criteria. In total, 21 students participated in March 2023, 18 of whom returned for follow‐up sessions in April 2023. As summarized in Table 1, participants identified with a range of sexual orientations and represented a diverse range of ethnic origins. Our international students came from a diverse range of countries and our home students represented a broad range of socioeconomic backgrounds. Participants included those living on or near campus and commuters and were studying a range of academic disciplines. All participants were aged between 18 and 25 years, predominantly at the lower end of this range.

**Table 1:** Summarizing participant demographics.

ID	Gender	Sexual orientation	Ethnic origin	Student status
P1	Female	Heterosexual	Asian or Asian British	International
P2	Female	Heterosexual	Black or Black British	Home
P3	Male	Heterosexual	Asian or Asian British	International
P4	Female	Bisexual	White or Caucasian	Home
P5	Female	Bisexual	Chinese or Chinese British	Home
P6	Female	Bisexual	White or Caucasian	Home
P7	Female	Prefer Not to Say	Chinese or Chinese British	International
P8	Female	Bisexual	Mixed	Home
P9	Female	Heterosexual	Black or Black British	Home
P10	Female	Bisexual	White or Caucasian	International
P11	Female	Heterosexual	Arab	International
P12	Female	Heterosexual	White or Caucasian	International
P13	Male	Heterosexual	Asian or Asian British	International
P14	Female	Bisexual	White or Caucasian	International
P15	Female	Queer	Asian or Asian British	International
P16	Female	Bisexual	Arab	International
P17	Female	Heterosexual	Mixed	Home
P18	Female	Heterosexual	Asian or Asian British	International
P19	Female	Heterosexual	Mixed	International
P20	Female	Gay/Lesbian	White or Caucasian	International
P21	Female	Heterosexual	Mixed	Home

### 2.2 Design and Setting

Four semi‐structured focus groups, each with three to six participants, were conducted in March 2023. Focus groups were adopted to enable consideration of the communicative context and to explore the content of shared and contested social understandings of the expectations of university social life (Flick 2025). An additional four focus groups were conducted with the same participants in April 2023 to validate the analysis of the first groups (Flick 2000). Each group lasted approximately 105 min and was conducted in‐person by two experienced researchers.

The topic guide for the first groups was designed to identify students' expectations of social connection before arriving at university. Students were presented with a diagram (see Figure 2) modeled on Bronfenbrenner (1979) ecological systems theory. Students were asked to use the diagram independently as a prompt to identify different social expectations, from different sources, that they held or had been aware of before university. This activity structured subsequent semi‐structured discussion around expected quantity and quality of social connections and activities at university, including romantic, academic, and familial relationships. Our questions intentionally did not direct participants to reflect on experience of loneliness. We sought to generate an unbiased insight into how expectations and experiences of social life might relate to loneliness. The topic guide for the second groups was informed by the initial thematic analysis of the first groups, exploring underdeveloped themes (see Appendix S1). Further, the second set of groups were used to explicitly address ideas and concepts that were “missing” from the initial focus group; this approach allows us to initially explore the ideas arising from participants without prompting or biasing the focus of discussion and to return to concepts prevalent in the literature. The second groups did this explicitly, explaining to participants that there are themes prevalent in the literature that they had not initially discussed and asking if these felt relevant to their experiences.

### 2.3 Analysis

A constructivist epistemological position was adopted, presupposing that data represent subjective perceptions of participant experiences and analysis reflects and contributes to the construction of meaning (Finlay 2021). Two reviewers (MP, NB) coded the transcripts separately using inductive reflexive thematic analysis (Braun and Clarke 2006, 2019; Bryant and Charmaz 2007). This involved adherence to the progressive phases of thematic analysis as identified by Braun and Clarke (2006, p.86), from data immersion, coding, thematic identification, and thematic review. A combination of semantic and latent codes was used to identify both the explicit meanings of expectations as expressed and to interpret the nature, source, strength, and consequences of these expectations. The reviewers subsequently conferred to iteratively review similarities and differences in coding structure and synthesize emergent themes. A reflexive approach was used, with consideration of how the researchers' prior knowledge and personal experience as current educators and student mental health researchers may have influenced interpretation. The refinement of themes was supported by critical review from the wider research team (JF, SC, EL, DF, SH, HS).

## 3 Findings

Our analysis identified three themes. While there were variations in participants' expectations, preferences and priorities, participants generally expected their university social life to be similar to school (Theme 2), contributing to a significant discrepancy between expectations and experience (Theme 1). Participants expected the university to play a far greater role in supporting friendship formation (Theme 3). Themes are elaborated, with participants quotes woven into the narrative flow to draw as closely as possible on the participants own phrasing.

**Theme One: “I never felt I got to experience uni life; it's literally the complete opposite to what I always associate uni with”: university social life is not as young adults expect.**

Many participants had high expectations for the number, frequency, strength, duration, similitude, and transitivity of friendships at university. Participants perceived a practical and emotional “*need to make friends”* [P8] and "*find good, valued friends who can like be there for you through the ups and downs”* [P3], because university "*does get hard sometimes and sometimes you just want someone to talk to”* [P2]. “*Day to day it is difficult doing things alone”* [P19].

By extension, whilst participants were “*expecting to have a lot more shallow encounters instead of close friends*“ [P4] they were “*seeking like a very small, bonded, friendship group”* [P17] and valued quality over quantity of friendships. Attributed to the increased proximity and opportunity to meet compatible people, there was a strong “*expectation that you're gonna find like 'your people'”* [P14]. Indeed, participants perceived that ‘*uni is where you make your friends for life and … [form] super meaningful connections’* [P5]. These expectations were not unanimously met; some participants “*came in with the expectation that if [they were] making friends, they would last, but that didn't happen”* [P19]. The transitory nature of social ties and the unexpected barriers to forming strong social relationships, including lack of structural support for socializing and the time and cost associated with engaging in social activities, frustrated participants' social needs, exacerbating feelings of social isolation and loneliness.

Alongside the quality and duration of new friendships, participants generally expected socializing to be frequent and at the forefront of their experience, "*expecting to always be doing something, I'd always be with people”* [P16] and “*basically never alone, always with people”* [P6]. A particularly prominent expectation was regular parties, which would be “*very drinking and clubbing heavy”* [P15] with “*lots of drugs, lots of alcohol”* [P2] and where “*the party life is like every single night”* [P2]. This expectation to “*go to uni, they are the best years of your life and just party every night”* [P17] compounded social dissatisfaction when not met. While some participants engaged with this aspect of social life, others felt shut out, either because of their choice not to drink or the time and cost associated with engaging, creating a feeling that they were “*missing out on core uni experiences, because when people talked about their uni experience, they would go out … and go clubbing… and for me, that wasn't the case … [and] it did, like, narrow the social circle that I had”* [P15].

In this way, many participants described a discrepancy between expectation and experience in the method and location of friendship formation. “I *went in thinking 'oh I'll make friends with people in seminars' and things like that. It didn't happen”* [P18]. A related mismatch was also apparent in relational transitivity. Whilst many participants sought “*a closely knit group of friends*“ [P21], they described unexpectedly having multiple disparate groups or individual friendships; "*It's not the same group of people doing the same thing. It's different groups of people doing different things with me”* [P9]. This absence of transitivity compounded the experience of loneliness given that “*when you have more individual friends than a group, you kind of find yourself stranded”* [P5]. In some cases, unanticipated heterophily further undermined relational transitivity and the sense of belonging to a group. Whilst many participants were “*expecting to meet people from different backgrounds and social classe*s” [P19], for others this came as a shock, “*I wasn't expecting it; it was very different because people are very diverse here”* [P9]. Diversity could challenge close identification with a group based on shared experiences, values, and beliefs given that “*you automatically feel closer if you are from the same background”* [P2].

Freshers' or welcome week represents an extreme point of discrepancy between expectations and reality. “*I expected I'm going to make so many friends, but I didn't make a single friend”* [P5]. Some participants expected to make long‐lasting friendships within the first week of university; "*it was like a big thing that everyone meets their friends through freshers but not after”* [P19]. This expectation compounded an anxiety that “*halfway through* [welcome week] *people have already found their friend groups so it's tougher to reach out and join that circle”* [P13] because “*if you are someone looking to make friends and everyone is already in the groups, that can be harder”* [P17]. For some, this meant there was a need to form friends immediately regardless of compatibility; "*it's like you meet your people in freshers and that's who you are stuck with for the rest of the time”* [P18]. Other participants “*thought that [they] would struggle more at first to like find people with similar interests and find people who [they] enjoy being with”* [P16] and therefore expected that “*it's probably going to take a while, like a couple of months to get to know people and form friendships that are like deep and last”* [P1].

Participants' expectations were being shaped by influences at the microsystem level, namely school teachers, the exosystem through older peers using social media, and the macrosystem, in the form of movies and television. Participants talked about parental advice, with parents noting that “*when you go to uni, you're going to find people that are actually your friends and actually like [you] for you, not just convenience*“ [P6]. Participants noted that “*a lot of people's parents met at uni*,” [P4] setting an implicit expectation to find long term romantic connections at university. Some, however, reflected that their parents had a very different university experience; if they had attended university, this was more than two decades ago or in a different country and understood as “*a completely different type of thing*“ [P14]. While not universal, some participants felt teachers “*were the ones that would plant the idea that [university is] the best years of your life”* [P16]. This was commonly a “*projection about [the teacher's] own university experiences”* [P18]. Other participants noted that in “*social media… and movies, like you kind of see two extremes and nothing in‐between. Like you party every night and you are just drinking and making a million friends. Or you are always by yourself and never going out.”* [P1]. Television was seen as unrealistically showing closely knit groups of students doing “*the same things every single day with the same group of people”* [P9]. Following older students from school on Instagram, gave the impression of a highly social environment as people would “*post a picture of a party or something”* [P3] and “*no one really posts them studying*,” [P2]. Other participants were drawing guidance from TikTok, a source of “*horror stories”* [P8]. The expectations for what university should be like were also being set in the first weeks of term where participants had the impression that “*like 90% of people [drink]”* [P12]. One participant explained, “*there were like events that were actually during the day that didn't center around drinking…”* but meeting people there, conversations were all “*like we were out drinking last night”* [P14].

In welcome week and beyond, participants described unexpected barriers to friendship formation, namely, time, travel, and cost. “*I had really high expectations for clubs … but what like I forgot to think about is that everything costs money”* [P10]. Other participants described how they “*would expect most societies to be at like one place*“ [P7] whereas “*the campuses are all split up and then events are happening in different places”* [P15]. As such, physical location of social events was a barrier to sociality; “*I live really, really, far away, so that's also the reason I kind of stopped societies”* [P11]. Unexpectedly time‐consuming academic demands compounded social dissatisfaction for participants that “*felt a bit overwhelmed with work and I felt like I was missing out on the social aspect*“ [P16].

During the transition to university, many participants described unexpected changes to the degree and/or immediacy of existing relationship qualities or dynamics. Whilst some participants expected and even desired the relocation to university to cause some relationships to “*drift apart”* [P16], others “*weren't expecting to have that shift … I thought my friends at home would be my real friends”* [P6]. For some, the transition led to a significant and unexpected re‐evaluation of the emotional value of these ties, reinterpreting previous connections as “*forced”* [P5] or “*superficial friendships*” [P19]. For others, “*coming to university actually helped me get closer to [old friends] because now that there's distance, you feel more obligated to be like, 'I need to be in contact with you, so you don't forget about me'”* [P15]. In this way, both the anticipation and the experience of relational decay was described to cause “*worry they would be doing stuff and then I would be the only one who can't join or can't participate”* [P16].

Likewise, some participants described the change in circumstances and increasing independence as creating an unexpected distance or barrier in their relationships with family; “*I changed a lot coming to [university], but back there everything is still the same, so there is a gap*” [P5]. Some students “*didn't expect the change to be quite so drastic*” [P14] in relational dynamic; “*I told my family or my dad that uni life is a little bit harder than I expected …. I got a pretty serious lecture like 'oh you have to do this on your own'”* [P8]. Participants similarly described unmet expectations regarding the sustainability of pre‐existing romantic relationships upon entry to university. “*I was in a long‐term relationship before starting uni. Thought we were going to be together forever…, And it lasted approximately 14 days at uni. The expectation is so different*” [P6].

**Theme Two: “I thought it was going to be like high school, that was my expectation”: students expect relationships at university to form and function like their relationships at school**

Multiple sources underpin and compound these discrepancies between expectations and experience. Emphasizing the absence of consistent or reliable information many students “*didn't really know what to expect*” [P19] and “*hadn't thought or set any expectations*” [P21] about university. In the main, participants that had siblings or social connections at university were better equipped to develop “*realistic expectations of what to do, what to expect, and whether it was going to be tough*” [P3]. Participants' previous social experiences at school or college significantly influenced their expectations of relationship formation and qualities at university. As such, many participants “*had high expectations… thinking it was going to be the same as school*” [P18] insofar that friendship groups would form based on shared proximity to a select group with a shared routine.

From school, secondary school, and sixth form you are literally forced into the same, like you have the form group, you meet every single day, and you are seeing the same 30 people every single day. So, you are kind of forced to make friends [P2].

Because many participants did not expect university to be as vast and disparate, they underestimated the associated difficulties in forming connections; “*Like when you go to school there is like sixty people in one class, but there are like two hundred plus people on just one [university] course*” [P9].

Whilst participants didn't “*imagine being actual friends with one of my lecturers”* [P12] and “*wouldn't want to be friends with them*“ [P6], there was a consensus that "*it is nice to have good relationship with the people that teach you”* [P19]. Drawing from experiences at school, most participants described “*expecting [relationships to be] like sixth form where teachers know your name, they know your life”* [P8]. Students thus expected that “*you'd have a lot more contact and a lot more interaction with lecturers”* [P1] and expected university “*to be exactly the same as it was at secondary school where your teachers know your name and know your personality. And when I got here that was very much not the case”* [P18]. Some participants had been warned that “*you won't have a personal connection with anyone in your faculty because there are too many students”* [P21]. While some participants felt “*the lecturers don't really care who you are”* [P2], generally, participants attributed the anonymity to large class sizes, with “*six hundred students, like I'm pretty sure they don't even remember my name*.” [P7]. This sense of anonymity was antithetical to feeling a sense of belonging and value at university; “*we are kind of like a bit of a blob. None of the tutors really know anything about us;”* [P6].

**Theme Three: “I feel like there was a lot of work on my part”: students expect the university to be more active in friendship formation and do not expect making friends at university to be so effortful**.

The unexpected differences between school and university required participants to “*put more effort in”* [P10] to make new friends than expected; “*It's not just like the friendships that form because you are around people”* [P10]. Whilst some participants “*did expect that it would have to be me”* [P14] to invest time and effort into forming and maintaining relationships, others expected “*to naturally form friendships*” [P9] through university classes, events and activities. Most participants expected induction events “*where they forced us to talk to each other”* [P2] and were therefore disappointed when “*they didn't force us to talk together: if we did talk, we would form friendships but we weren't exactly forced to, so if you didn't really start conversation or initiate anything, you would kind of go the whole day without making any friends*” [P1].

This unanticipated effort and energy associated with putting oneself “*out there in a way that I'm not used to doing”* [P9] was described as “*exhausting in every way*“ [P10] and “*a huge drain of social energy*“ [P1]. This unexpected effort compounded the discrepancy between expectations and experience of the frequency of socialization: “*I came here expecting to always be doing something, I'd always be with people and have like, no, free time. But then after the first day I was like ‘no, I'm going to rest for a bit”* [P16].

Participants generally described increased autonomy over social activities and relationships at university because “*you are independent. It allows you to make choices and decisions for yourself whereas like a lot of the choices when you are in school, your parents would have to say something”* [P3] or “*social spaces and times were dictated by school”* [P19]. With this increased autonomy, participants experienced unexpected effort to maintain an active social life; “*if I want to interact with people, I have to organize it*” [P10]. In this way, many participants did not expect independent time management at university to be as challenging or to have such a significant implication for social relationships; “*you really have to be, like, very intentional and very like organized with meeting up with people”* [P14].

## 4 Discussion

While our findings support a cognitive discrepancy model of loneliness, young adults described complex and heterogeneous dimensions of expectations, highlighting many different ways in which expectations and experience can be misaligned. Reflecting developmentally relevant priorities, belonging to a close‐knit group of friends was prioritized relative to having individual friends. Participants were overwhelmingly using their school experience as a frame of reference to shape their expectations for university, especially in relation to the effort required to form relationships. This amplified a disparity between expectations and experience (Perlman and Peplau 1981).

Students were looking for lasting, positive and significant interpersonal relationships (Baumeister and Leary 1995; Baumeister and Leary 2017). In the early weeks at university, the lack of these connections was unsettling. Further disquiet arose from social comparison; the impression that others were forming friendships or had strategies to help them make friends, primarily through drinking and partying. Here the perceived discrepancy between expectation and experience seems to be felt more acutely because of heightened sensitivity to peer comparison (Albert and Steinberg 2011; Foulkes and Blakemore 2016; Somerville 2013).

Young adults described multifaceted expectations for social connection, relating to the volume, frequency, strength, duration, similitude, and transitivity of relationships. While participants were looking for stable, strong, and meaningful relationships, group dynamics were important, with value placed on close‐knit groups of friends and transitivity. Consistent with literature on distinct social and emotional relational needs (Qualter et al. 2015; Weiss 1974), in addition to desiring meaningful connections, students expected frequent socializing, and an intensely social experience.

As in previous studies, many students held high expectations for sociality at university (Balloo 2018; Balloo et al. 2017; Hughes and Smail 2015), and reported that university life did not meet these expectations (Barron and D'annunzio‐Green 2009; Borghi et al. 2016; Briggs et al. 2012; Brinkworth et al. 2009; Crisp et al. 2009; Keup 2007; Smith and Wertlieb 2005). Again, differentiating between social and emotional relational needs, while there was appreciation of the time and effort required to form meaningful connections, students seemed to expect a relatively effortless intensely social experience. Whilst the sample was disproportionately international students (HESA 2023) and international students may be at elevated risk of loneliness (Sawir et al. 2008; Wawera and McCamley 2020), interestingly these expectations and experiences were found to be largely consistent among home and international students. Both groups were looking for strong interpersonal relationships, expected an effortless intensely social experience and experienced distress from social comparison.

Expectations around the immediacy, strength, and homophily of new connections may be compounded by media and university marketing messages (Bates and Kaye 2014). Prospectuses depict university as a social idyll, guaranteeing “experiences that form a sense of compatibility and belonging” (Mogaji and Yoon 2019, p.1561) However, participants made limited reference to using information from universities to shape their expectations for student social life. If anything, it would appear that universities are doing very little to set students' expectations, realistic or otherwise. Rather, they are drawing information from parent and schoolteachers, drawing from their own experience, older peers sharing their experiences via social media, and mass media via films and television. In the absence of information about university life, students are modeling their expectations of university on their experiences of school.

As in previous studies (Scutter et al. 2011), students described having unclear or inconsistent expectations of university life. Widening participation and growing numbers of international students have increased diversity in student expectations for university (Barron and D'annunzio‐Green 2009; Bolton et al. 2024; Money et al. 2017). Where different student groups have different access to information, networks, experiences, and resources to develop expectations of university (Balloo et al. 2017; Barron and D'annunzio‐Green 2009; Bathmaker et al. 2013), we risk inequalities in social capital translating into heighted risk of loneliness (Read et al. 2020).

Echoing previous evidence, this study identified particularly striking discrepancies between students' expectations and experiences for academic support (Bates and Kaye 2014). Shaped by an expectation that it would be the same as school (Money et al. 2017), many students expect regular personal communication from academic staff (Balloo 2018; Barron and D'annunzio‐Green 2009; Borghi et al. 2016; Brinkworth et al. 2009; Crisp et al. 2009; Tomlinson et al. 2023). While students do not expect lecturers to be part of their social circle, they do expect them to know their name and be proactive in supporting them to build a sense of connection with their peers.

### 4.1 Implications

In illustrating the complex and multifaceted nature of expectations, our findings suggest that a full understanding of loneliness at this transition point needs to reach beyond simple conceptualizations of the quantity and quality of social relationships. The distinction between social and emotional loneliness is important for young adults and understanding expectations and experience of group dynamics alongside individual relationships is consequential for subsequent practice.

#### 4.1.1 Our Research Suggests Several Practical Implications

##### 4.1.1.1 Transition Into University

Loneliness may be reduced through support to set realistic expectations at times of transition. Opportunity to develop social skills before transitioning out of school may familiarize young adults with the challenges and effort involved in making and sustaining friendships. Schools may be well placed to support some of this preparation; arguably workshops and mentoring could be provided and may have particular benefit for disadvantaged pupils (Holland 2010; Redeker et al. 2012). Universities may benefit from providing a more realistic and informed picture of university social life, through existing induction events and taster sessions (Cage et al. 2021), as these initiatives can minimize the incongruity between expectations and experience (Briggs et al. 2012; Brinkworth et al. 2009) and support social integration (Pennington et al. 2018).

##### 4.1.1.2 Social Integration

Students expect the university environment, classroom structures and timetables to support friendship formation and socializing. Considerable impact may be realized by reviewing university timetabling to assess whether schedules maximize opportunities to consistently spend time with the same peers and allow time for socializing between classes. Preliminary evidence indicates that peer‐led social activities holistically embedded in the curriculum are acceptable, accessible, and broadly effective in a student population (Cheng et al. 2022). Leaving social aspects of university life to students to organize appears to result in a substantial focus on drinking and partying. Universities may need to take greater responsibility for organizing social activities to provide an alternative to the impression of a drinking culture (Priestley et al. 2022).

##### 4.1.1.3 Personalized Academic Interactions

Drawing from their experiences at school, students expected to be known by name. While students recognize that this is an unrealistic expectation with large cohort sizes, they place a high value on being known (Bosch 2024; Cooper et al. 2017). This underlines the importance of taking advantage of opportunities for academic staff to connect with students by name, when possible, and looking for opportunities for consistency in pairing students and teaching assistants to maximize the chance of developing a more personal relationship.

### 4.2 Strengths, Limitations and Future Directions

We were able to take a two‐step approach to data analysis, taking our initial thematic analysis to our participants, to involve them in discussions of the theme development. Whilst power dynamics between student and researcher may have prevented explicit critique of initial themes, the process enriched the analysis by enabling focused clarification and exploration of underdeveloped responses, such as family relationships.

The diversity of the sample provides insight into the expectations of young adults from a wide range of backgrounds. However, the study focused on a single large London research‐intensive university, with a predominantly female sample. As such, the findings around experiences may not be representative of the student experience across the UK. Concerns around the costs, time, and complexity of socializing may be especially acute for students studying in London and the pressure to focus on academics may reflect the highly academic institution. The lack of connection with academics may be less of a concern at more teaching‐focused‐ institutions. There are important gender differences in social context that our study has not explored (Barreto et al. 2021; Flynn et al. 2017). Future research could benefit from examining the expectations and experiences of a wider sample of young adults during different transition points and further interrogating the differences between demographic groups (Nadelson et al. 2013), particularly with regard to the relative strength and source of different expectations (Tomlinson et al. 2023).

While we focus on young adults, given their increased developmental risk of loneliness, approximately 25% of all undergraduate students are 25 years old or older (HESA 2023). Mature students come university with different goals, priorities and expectations (Saddler and Sundin 2020; Shanahan 2000). Their experience of social life at university may be very different to their younger peers, with unique challenges arising from the perceived norm of undergraduates being young adults (Gregersen and Nielsen 2023; van Rhijn et al. 2023).

Data collection asked participants to recall expectations before attending university. Whilst this can offer benefits in reflecting on the discrepancy between expectations and experience (Money et al. 2017), the data may be subject to emotional and recall bias (Talari and Goyal 2020). “Longitudinal research tracking the links between expectations, experience and satisfaction would provide universities with key information” on points of intervention for different student groups (Balloo 2018, p.2259).

Focus groups provide an opportunity to explore shared and contested understandings of a phenomenon within a socio‐cultural context but may discourage open disclosures around experiences relating to sensitive topics, such as loneliness. The data in our study consequently concentrated on shared and contested expectations and experiences of social life at university and does not provide in‐depth insights into individual experiences of loneliness. Future work using one‐to‐one interviews will help elucidate individual experiences of loneliness.

## 5 Conclusion

While our findings support the cognitive discrepancy model of loneliness, they also underline the complexity of expectations for and experience of social connection. Understanding and addressing loneliness among young adults may require identification and management of potentially unrealistic or inaccurate social expectations, particularly those generalized from the school context.

## Author Contributions

Conceptualization: M.P., H.R.S., D.F., J.F., S.H., M.I., and N.B. Data curation: M.P. Formal analysis: M.P. and N.B. Funding acquisition: D.F., E.L., J.F., S.H., and N.B. Investigation: M.P., H.R.S., and J.F. Supervision: N.B. Writing – original draft: M.P. and N.B. Writing – review and editing: M.P., H.R.S., M.I., D.F., E.L., S.C., J.F., S.H., and N.B. This study was conducted in accordance with the Declaration of Helsinki on research ethics and was registered with the King's College London Research Ethics Committee (MRA‐22/23‐34142).

## Supporting information

Supporting information.

## Data Availability

The data that support the findings of this study are available on request from the corresponding author.
