# Supplementary material for: “No One Told You Life Was Gonna Be This Way”: A Qualitative Exploration of Friendship Expectations and Reality in University Life
Source: J Adolesc. 2025 Apr 4;97(5):1225–35. doi: 10.1002/jad.12489 (PMC12217411; doi:10.1002/jad.12489)
Supplement: Supplementary file 1 — Supporting information. [file JAD-97-1225-s001.docx]

**Appendix One**

**Topic Guide**

**Specific Questions**

- **1A. Frequency**
  - Does this capture what you shared with us in relation to the amount of time/ how often you expected you would be with friends and other people at university as well as time spent alone? Does this feel like an important and distinct part of your expectations of university?
  - We talked a lot in the groups about an expectation of partying every night. Was this just an expectation of freshers week or throughout your whole time at university?
  - Is it helpful to distinguish how often you expected to see people you know beyond just friends. In some of the groups we talked about regularly seeing and spending time with people at school that aren’t friends and in another group we talked about how on campus you never see the same people again. Is this an important distinction in what you expected? Should we have an item like ‘I expect to see people I know every day at university’?
- **1B. Duration**
  - Do these items capture what you shared with us? Was the length of friendship an important distinction you thought about when coming to university? Or was it just the strength of the friendship that is important, and friendship length is a reflection of that?
  - Do you think all students will expect to make friends for life and/or have friends that come and go? Or is there some variability?
  - In one of the focus groups it was mentioned that there is a pressure to make friends for life at university because it is harder to make friends after university. Is this something that others were aware of? Do you think the ‘because it will be hard to make friends after university’ is an important and distinct expectation to capture?
  - We talked a bit about forming friendships that end because people fall out, although some people said they had never really thought about or experienced this. Is it important to differentiate friendships that naturally come and go with friendships that end because of conflict? Was this a distinction that you had in your mind before coming to university?
- **1C. Strength**
  - Do these items capture our discussion about how close you expected to be with university friends, including in relation to other pre- university friends?
  - Are ‘close friendships’ the same as ‘meaningful friendships’? What are ‘shallow encounters’ and are these the opposite of close/ meaningful friendships?
- **1D. Size**
  - Does this capture what we discussed in relation to the number of both acquaintances and friends you expected would be in your social circle at university, and whether you would be spending a lot of time with one small group or meeting and spending time with lots of different people?
- **1E. Homophily**
  - Does it make sense to differentiate between meeting people similar/ different to you and making friends with people similar/ different to you? Was this an important distinction in your expectations? Did you expect there to be a diverse range of people at university but you would only be friends with people with certain things in common to you? Was the important thing that people would be similar/ different to you, or more similar/ different to you than you had previously had opportunity to meet/ make friends with?
  - Were these expectations mainly around similair backgrounds and characteristics (e.g. same university) or are there more factors that you think about being similar or different to a potential friend on? Should it be something broader like ‘people in common with me’? Some people talked about making friends from ‘similar backgrounds’ and some people talked about making friends with people with similar life experiences – are these the same thing?
- **1F. Transitivity**
  - Transitivity just means how interrelated your social connections are. Does this capture our discussion about having different and separate groups of friends or having one interconnected group of friends? Do all students expect to have a friendship ‘group’ at university or is important to distinguish the expectation to make one or two strong friendships? And have we captured this?
- **1G. Change**
  - We talked a lot about expectations for changes to prior relationships depending on the strength of those relationships. How can we better capture this in the items? Should we be asking about the scale of change? Did you have separate expectations for the number of relationships you expected to maintain, and the strength of the relationships you expected to maintain, or were these interrelated? Have we captured the expectation to not make many new friends because you are satisfied with old friends?
  - Have we captured our discussion about some friendships strengthening because of having new stories to share and making more effort?
- **1E. Immediacy**
  - Do these items capture our discussion about how quickly you expected to make friendships of differing strength and the expectation that everyone meets their friends in freshers week and not after?
- **1F. Online**
  - Should this be a separate theme? Does it make sense to specifically think about forming and maintaining relationships online (e.g. I expect to maintain my old friendships online; I expect to use social media to meet new people) or is this so embedded into how we make and maintain relationships that it doesn’t make sense to ask it separately?
- **2A. Places**
  - Does this capture our discussion of how you expected to meet, make, and maintain friendships at university?
  - How can we find better wording than ‘outside of the university environment’ to capture our discussion of making friends at work or in other community places (e.g. church), including with people not at the university?
  - Is it helpful to be saying ‘most’ (e.g. I expect to make most of my friends on my course). If we said ‘some friends’ or just ‘friends’ would there be any variability or would all students agree that they expect to make some friends in these places?
  - Is it important to distinguish expectations of drinking from clubbing (e.g. drinking in other environments; clubbing not drinking)?
  - Is it helpful to differentiate the different ways that students make friends on their course? Was this an important distinction in your expectations? E.g. ‘I expect to make friends with the students sitting next to me in lecture halls’; ‘I expect to make friends with students through group work in classes’; ‘I expect to make friends through working with my peers in seminars / practical classes’. Or is this overkill?
- **2b. Effort**
  - Does this sub-theme and the items capture our discussion about making choices and investing time and effort into making/ maintaining friends at uni compared to school, where you make friends by being with the same people?
  - Does this capture your expectations for what the university will do and university events/ activities to help you make friends? Should we be making a distinction between expectations of what the university will do at the start and then throughout? Is it helpful to be more specific about the types of social events (e.g. I expect my course to put on events to help me to get to know my peers on my course). In a couple of the groups we talked about an expectation that there would be university wide events. Was this just an expectation that there would be more/ bigger events as a chance to meet more people? Or was this more about the expectation of the university/ academics being a sense of wider connection and identity to the university?
  - Have we captured our discussion around the effort involved in forming friendships. E.g. ‘I expect that meeting new people will be tiring/ drain my social energy’. Is this important to differentiate?
- **3. Expectations of Relational Qualities and Functions**
  - This theme tried to capture all our discussion about what a good friendship would look like.
- **3A. Shared Values**
  - This sub-theme tries to capture our discussion of the expectations for forming friendships with people with similar personal values/ qualities to you, whereas homophily tries to capture our discussion of making friends with people from similar/ different backgrounds to you (so you could have a similar background but different values to someone). Have we got this right? Is this important to you? Is this clear in our terminology? Is ‘shared values’ the same as what we described about vibing/ gelling with someone? How can we use better/ clearer wording than ‘values’ to make this distinction? Is it more about a shared understanding than shared values?
- **3B. Shared Activities**
  - This sub-theme tries to capture an important distinction in our discussion of friends being people we choose to spend time with when we don’t have to, with shared interests/ activities forming the basis of these friendships (but that you can still be friends even if you have different interests as long as you have shared values). Have we got this right? Do similar interests always include doing the same activities and hobbies? Or do we need to differentiate this? Was our discussion of shared lifestyle as important in friendship particularly in relation to activities (e.g. clubbing; sport) and values (e.g. valuing work) or is it something separate for a different reason?
- **3C. Informational/ Emotional/ Practical/ Instrumental Support**
  - This subtheme tries to capture our discussion of the different types and purposes of different relationships you have with different people at university [e.g. we talked about flatmates and course mates as not always being ‘friends’ but something different]. Do these categories reflect the way you experience the nature of different relationships and your expectations of different relational functions when coming into university?
  - Does ‘informational support’ capture our discussion of mentorship from students in older years and making friends that help you navigate living in a new place?
  - What do you understand by practical support? Does this capture our discussion about people who would give physical help on your course or lend you something in your accommodation? Is there better terminology we could use?
  - In a couple of groups we talked about how friends on your course can add to academic pressure. How can we capture this? Is this about the characteristics of the person or the quality of the relationship?
  - We talked about relationships with staff as potentially being beneficial for career. Do we need to differentiate the type of relationships here? Did you/ do others expect to make connections with peers that could help you in your career? Or is it only staff?
  - Is it important that the wording of these items shows that these relational qualities/ functions are mutually supportive? Should we ask separately about relationships where I support you, you support me, and we support each other. Is feeling needed distinctive from being supportive? And was this important to you?
  - In some of the groups we talked about needing/ not needing to make friends. What does need mean? What are the attributes of friendships that would mean it is needed or not needed. Is this the same as making friends being a priority?
- **3G. Independence & Belonging**
  - We will discuss these more separately.
- **3H. Isolation/ Conflict**
  - We talked in one focus groups about an awareness of good friends arguing and being bullied, and then in another about a general sense of tension with people that hold different views to you. Does it make sense to ask about tension and conflict together? Or would you have completely different answers depending on which you were thinking about?
  - Does this capture our discussion about tension/ conflict in accommodation? Or is it helpful to specify this more clearly?
  - In one focus group, someone talked about their course being really competitive and this limiting the friendships they were able to make. Are other people aware of this? Is this because it changes how you see and interact with people? Or is this because it make you feel you don’t have time to make friends? Should we have an item like ‘I expect to feel the need to compete with my peers academically’?
  - How important was the expectation of peer pressure when coming to university and have we captured this (e.g. in the emotive subtheme). Is it helpful to differentiate different sources of peer pressure (e.g. pressure to drink, hook up etc).
- **4/5. Personality/ Priority/ Multiple/ Distorted/ Emotional**
  - Do these items capture our discussion about how different people will value the social life at university differently and have different expectations of it? And getting the right balance between different expectations? Do the ‘selective’ items feel value laden?
  - Did you expect to experience FOMO? Did you expect to be able to attend all the events / activities that you would like to engage with?
- **6A. Practical**
  - We talked a lot about not really expecting to think about travel time to get to social activities, assuming everything would be close to your accommodation, and how this restricts the social activities that you can take part in. Does the single item ‘I expect it will be easy to access social events and opportunities at university without having to travel’ capture all of this? Or do we need to ask more directly ‘I expect that travel logistics will be a barrier to attending social events and opportunities at university’?
- **6B. Institutional**
  - We talked about London in terms of making it difficult to travel and actually meet people. But is there something more about the size of London (not just the size of Kings) that makes it difficult to build a sense of belonging and connection? Was this something you expected? How can we capture our discussion about the different implications of campus and city universities on the social experience? Again, was this just about the opportunity for social connection or the feeling of connection/ belonging too?
- **7. Romantic**
  - Do these items capture all our discussion about expectations about romantic relationships? Have we missed or misrepresented anything that you/ others expected about romantic relationships at university? Is the opportunity to form more romantic relationships also about having more freedom to do so, and is this something important we need to differentiate?
- **8. Academic**
  - Do these items capture all our discussion about expectations for relationships with academic staff?
  - Have we missed a sub-theme/ item about the way you expected to be taught (e.g. lectures, seminars) and the impact of this on your relationships with academic staff (e.g. I expect to have teaching in small groups enabling me to make friends with my classmates; I expect to have many face-to-face teaching hours, providing lots of opportunity to make friends with classmates)? Should we have an item about the number of academic staff you expected to be taught by/ interact with (e.g. I expect to see my lecturers every day/ every week)? Is this important and a different expectation to school? We talked about the time and other commitments university staff have? Is this an important and distinct expectation or is this just relevant in terms of actually being able to form a personal relationship?
  - Would everyone expect and has everyone experienced academic staff that they could go to if they needed help with their course? Or do we need to differentiate this?
- **3. Missing Items**
- When reviewing the literature, we identified a few themes in relation to student social expectations which did not come up in our analysis of the first focus groups. These will form the basis of discussion with students to understand whether they are important to student expectations and experience and/or whether we missed or misunderstood their relevance.
- **3a. Family**
- We talked about family in relation to forming expectations of university and we talked about changes in social networks but we didn’t really talk about expectations of whether and how relationships with family would change at university? Is this important? Did you expect your relationship with your family to stay the same? Have less contact with them? More contact with them? How important is it to have support from family when transitioning into university? (How)/ does this sit alongside any desire for independence? Do family support you to become independent or do you become independent by separating from family?
- **3b. Local Community**
- We talked a bit about friendships outside of university and how location influences friendships, but not really about the importance of developing a sense of connection to the local community. Is that important or something you thought about before coming to university? Did you think/ care about feeling like part of the local community in the area that you lived or just think about relationships with students at the university?
- **3c. Identity, Role, Responsibility, and Independence**
- We talked a tiny bit in some of the groups about expecting to have increased independence at university and this shaping expectations for social relationships (e.g. more independence from parents to socialise or form romantic relationships). Did you expect to have independence at university? Did you expect to have more independence than at school/ college? How important was independence as an expectation of university and in shaping the social experience? Did you want university to be a time of independence to explore your identity and find yourself? And was this intwined with the social relationships you wanted to form? Do you think having more independence at university effects the social experience at all or is this something different?
- 3d. **Belonging**
- We talked about the features, qualities, and characteristics of social relationships with peers at university but not really in terms of belonging to the university. Was this something important to you before coming to university? Some of the groups talked about feeling anonymous at university and some groups talked about an expectation of university-wide events that build identity but which was not met. Did you have a sense of wanting to feel a valued part of the university community/ sense of belonging to the university campus/ sense of university spirit? Or were you mainly just concerned with actual relationships and friendships with individuals and smaller groups? Or are these the same thing/ lead to one another? Did you have expectations to feel a sense of belonging or identification to a specific group such as a society or your academic course, and is this different to a sense of belonging to the university?
- **3e. Identification**
- How much does identifying with one smaller group matter? Can you still feel a sense of belonging if you are part of a small group even if you feel excluded from the wider group?
- **3f. Self-efficacy and confidence**
- We talked about different students prioritising their academic studies and social interests differently because of different preferences. Is this a personal preference or is this because certain students have more confidence academically? Do some students feel they can socialise more because they are more confident that they can manage their work? How important is self-confidence in shaping and understanding student social expectations.
- **3g. Other Adults**
- We talked about relationships with academic staff. Did you have any expectations of relationships with any other older adults at university? Was this something you expected? Or has this been something that you experienced and which has been important? Do older adults support you to become independent or does independence come from separating from older adults.
